# Daily artificial gravity partially mitigates vestibular processing changes associated with head-down tilt bedrest

**DOI:** 10.1038/s41526-024-00367-7

**Published:** 2024-03-12

**Authors:** G. D. Tays, K. E. Hupfeld, H. R. McGregor, N. E. Beltran, Y. E. De Dios, E. Mulder, J. J. Bloomberg, A. P. Mulavara, S. J. Wood, R. D. Seidler

**Affiliations:** 1 Department of Applied Physiology and Kinesiology, University of Florida, Gainesville, FL USA; 2 KBR, Houston, TX USA; 3 German Aerospace Center (DLR), Cologne, Germany; 4 NASA Johnson Space Center, Houston, TX USA; 5 Norman Fixel Institute for Neurological Diseases, University of Florida, Gainesville, FL USA

**Keywords:** Neuroscience, Systems biology

## Abstract

Microgravity alters vestibular signaling and reduces body loading, driving sensory reweighting. The unloading effects can be modelled using head-down tilt bedrest (HDT). Artificial gravity (AG) has been hypothesized to serve as an integrated countermeasure for the declines associated with HDT and spaceflight. Here, we examined the efficacy of 30 min of daily AG to counteract brain and behavior changes from 60 days of HDT. Two groups received 30 min of AG delivered via short-arm centrifuge daily (*n* = 8 per condition), either in one continuous bout, or in 6 bouts of 5 min. To improve statistical power, we combined these groups (AG; *n* = 16). Another group served as controls in HDT with no AG (CTRL; *n* = 8). We examined how HDT and AG affect vestibular processing by collecting fMRI scans during vestibular stimulation. We collected these data prior to, during, and post-HDT. We assessed brain activation initially in 12 regions of interest (ROIs) and then conducted an exploratory whole brain analysis. The AG group showed no changes in activation during vestibular stimulation in a cerebellar ROI, whereas the CTRL group showed decreased activation specific to HDT. Those that received AG and showed little pre- to post-HDT changes in left vestibular cortex activation had better post-HDT balance performance. Whole brain analyses identified increased pre- to during-HDT activation in CTRLs in the right precentral gyrus and right inferior frontal gyrus, whereas AG maintained pre-HDT activation levels. These results indicate that AG could mitigate activation changes in vestibular processing that is associated with better balance performance.

## Introduction

Following spaceflight, astronauts return to Earth with transient deficits in their balance and mobility; these can last for several weeks before performance returns to preflight levels^1,2^. This is thought to be largely due to altered vestibular signaling and multisensory re-weighting that occur in microgravity. Vestibular inputs in a microgravity environment, particularly from the otoliths (which detect linear accelerations and head tilt relative to gravity) are down-weighted because the signals are unreliable in the absence of gravity^3,4^. Once an astronaut returns to Earth, adaptive responses become maladaptive in the presence of gravity, temporarily hindering locomotion and balance performance^5^. Studies have also shown vestibular neural changes either during flight or post-flight, such as reduced in-flight electroencephalography alpha power localized to the vestibular, motor, and cerebellar brain regions^6–8^. In addition, a case study reported decreased vestibular resting state network connectivity postflight^9^. Our own recent work has supported the sensory re-weighting view, based on analyzed functional magnetic resonance imaging while astronauts received vestibular stimulation pre- and post-flight^3^. Typically, on Earth, vestibular stimulation elicits brain deactivation in cross modal sensory regions^10,11^. However, following ~6 months of spaceflight, among 15 astronauts, we identified widespread pre- to post-flight reductions in this brain deactivation across sensorimotor, frontal, temporal and occipital regions; we interpreted this increased deactivation as suggestive of upweighting of somatosensory and visual processing during flight (when vestibular inputs are unreliable)^3^. Further, we identified a brain-behavior correlation between pre- to post-flight activation change in visual and multisensory integration brain regions and pre- to post-flight change in balance. Those that had greater reductions in deactivation of these brain regions from pre- to post-flight maintained better balance performance^3^. In summary, spaceflight impacts the brain’s processing of vestibular stimuli and sensory weighting for motor control.

Long duration head-down tilt bedrest (HDT) has been repeatedly used as a spaceflight analog to model physiological changes that occur in microgravity. Participants lie with their head 6° below their feet, resulting in headward fluid shifts, axial body unloading and other physiological effects of microgravity^5,12–17^. HDT does not directly affect vestibular inputs, however it is thought to initiate sensory re-weighting which will indirectly affect processing as the vestibular nuclei also receive proprioceptive inputs from the limbs^18–20^. During bedrest, subjects are deprived of higher frequencies of linear accelerations that are associated with locomotion^21^. If vestibular or somatosensory inputs from the limbs are disrupted, the central nervous systems may upregulate other sensory systems to compensate and maintain performance^22–24^. During HDT typical somatosensory inputs to the foot sole are removed and vestibular processing appears to be altered as vestibular cues are upweighted, or relied upon more heavily^5,25^. Performance of behaviors that depend upon the vestibular system and multisensory integration, such as functional mobility and postural stability, have been shown to decrease following HDT^5,13–17,26^.

We have previously demonstrated that HDT affects the neural correlates underlying vestibular processing^27^, and further, HDT in conjunction with elevated CO_2_ (such as occurs in the enclosed environment of the International Space Station (ISS)) affects vestibular processing^25^. We found that HDT results in increased activation in portions of the insular, frontal, and parietal cortices during vestibular stimulation, suggesting that more brain resources may be required to process vestibular information during HDT^27^. When investigating the added effects of CO_2_ to HDT, we identified increased activation from to pre-HDT to post-HDT in the left inferior temporal gyrus, right superior occipital gyrus and brainstem in those that had increased levels of CO_2_ relative to HDT alone^25^. Further, greater deactivation in various regions was associated with sustained behavioral performance in mobility and balance tasks^25^. That is, participants that had greater deactivation pre-to-post HDT had the best behavioral performance from pre-to-post HDT. Overall, HDT has been shown to influence vestibularly mediated, multi-sensory behavior and brain activation patterns that underlie vestibular processing.

Countermeasures for post-flight physiological and functional changes have been under investigation for many years. Since many different systems are affected by spaceflight (muscle, bone, cardiovascular, neural, etc.)^28^, the ideal countermeasure would be integrated to target many systems at once. Short arm artificial gravity (AG) has been proposed to provide multisystem benefits^29,30^. In spaceflight, the otoliths cannot signal head tilt, there is bone and muscle loss, proprioceptive sensors receive reduced stimulation, and there is cardiovascular deconditioning^4^. On Earth, AG can be applied along the long axis of the body via centrifugation. The participant lies in a supine position and is spun to create 1 g at their center of mass. This protocol has previously been investigated with participants undergoing 5 days of HDT and receiving two different AG exposure protocols. AG applied intermittently (in 6 bouts of 5 min) was shown to mitigate decreases in orthostatic tolerance due to HDT^31^. Further, we used the same AG protocol in conjunction with NASA and ESA (Artificial Gravity Bed Rest-European Space Agency; AGBRESA) for a 60-day campaign of HDT to investigate the counter-active effects of AG on sensorimotor and cognitive performance^32^. We found that AG may serve as a countermeasure for balance and mobility deficits that occur with HDT, and further, during centrifugation participants were able to perform better on a cognitive task than controls who performed in bedrest^17^. This was supported by other work, finding that intermittent AG partially mitigated the deterioration in sway path and velocity, as well as sway frequency power^33^. However, Clements et al.^32^ analyzed balance performance in the AGBRESA campaign as well, and identified no mitigation effects of AG on balance performance pre-to post-HDT. In the same campaign, we found that AG increases neural efficiency during sensorimotor adaptation tasks^34^.

In the current study, we tested vestibular processing in the same manner as our previous HDT^25,27^ and spaceflight studies^3^. Our primary aim was to examine if AG applied along the long axis of the body mitigates vestibular processing changes that occur with HDT. We hypothesized that control participants would show greater pre- to post-HDT changes in brain activity during vestibular stimulation compared with those receiving the AG intervention. Further, we predicted that individual differences in brain changes would be correlated with decreases in balance and mobility from pre- to post-HDT.

## Results

We initially examined for statistical differences in brain activation between the two AG groups (continuous and intermittent) and found none; thus, we pooled them together into a joint AG group.

### Baseline main effect

**Fig. 1:**
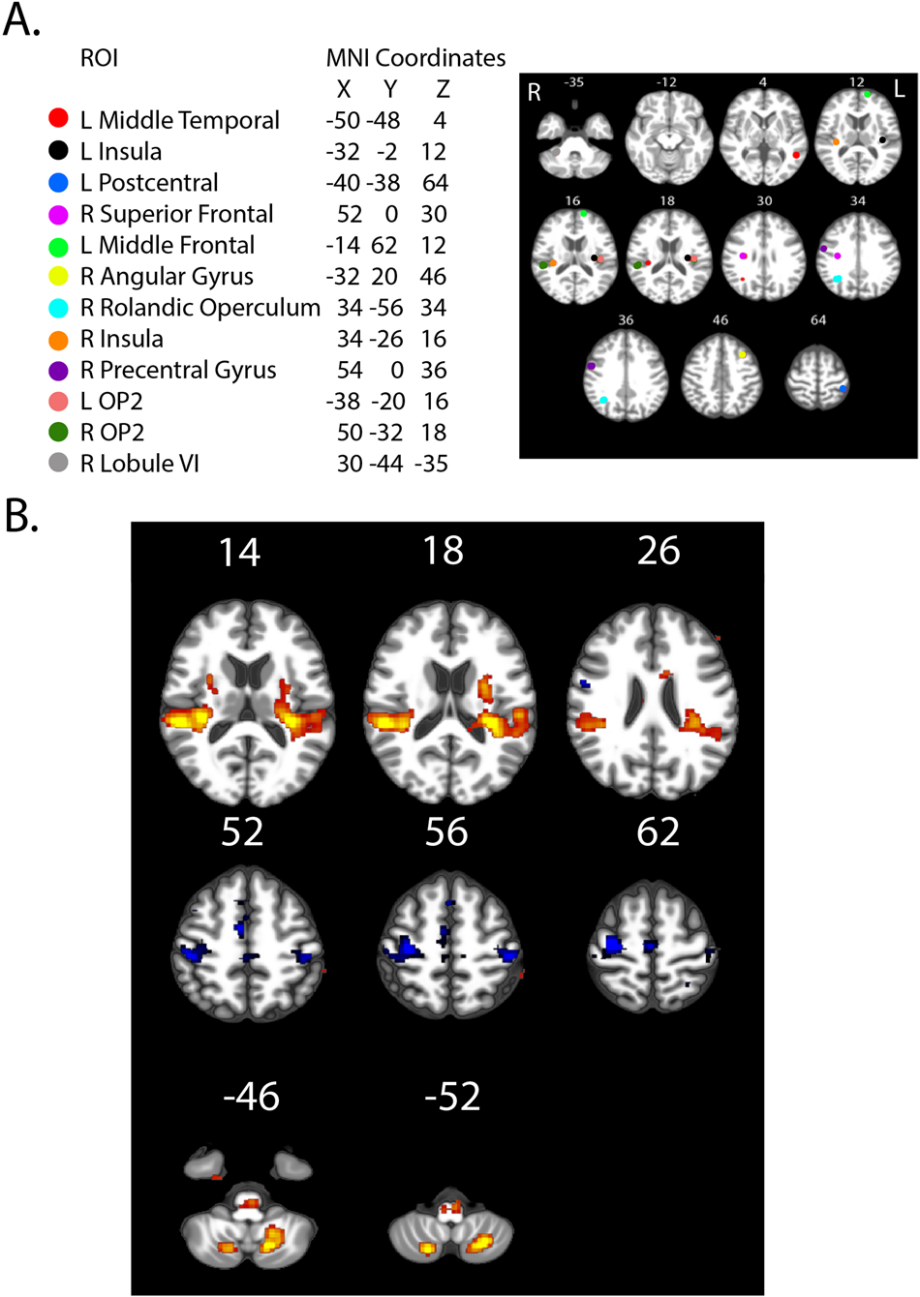
Pre-HDT VEMP. **A** ROI spheres placement. **B** Main effect results of vestibular stimulation compared to rest at the pre-HDT time point (across all participants, *n* = 24), all participants were included in one group as they had not entered the HDT environment. Red/orange indicates regions activated during vestibular stimulation compared with rest, blue indicates regions that are deactivated during vestibular stimulation compared with rest. The numbers in the figure refer to the Z level of the depicted slice. The left side of the image is the right side of the brain.

First, in order to assess the baseline activation of the VEMP task, we conducted a main effect analysis of activation compared to rest pre-HDT. Like our and others’ previous work^3,10,11,25,35,36^, vestibular stimulation versus rest resulted in activation in the right and left rolandic operculum, right temporal supplementary region, right hippocampus, right para-hippocampus and cerebellar lobule 8 at the baseline condition (prior to entering HDT). Further, we observed deactivation in somatosensory and motor cortices (Fig. 1), as we expected^37–40^. This analysis was conducted with all subjects in one group, prior to entering HDT all participants are treated similarly and naïve to the group designation.

### ROI based analyses

**Fig. 2:**
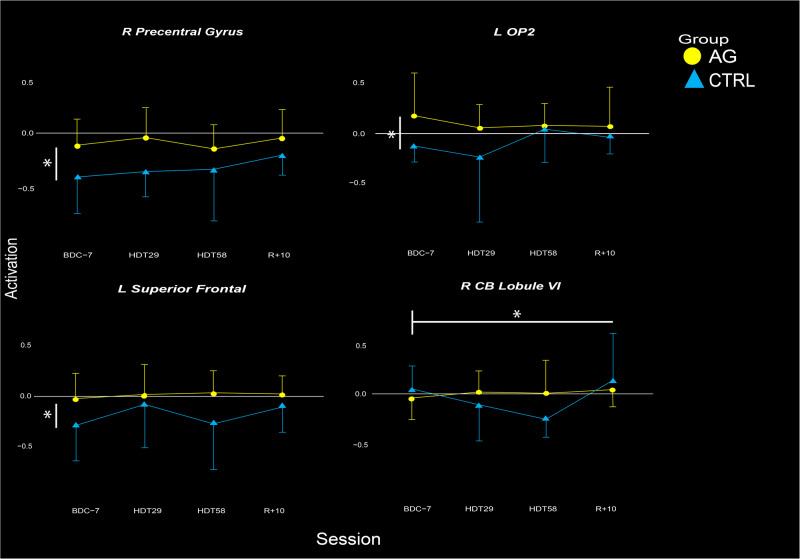
ROI Activation. Activation patterns in the four ROIs that showed significant changes or differences reflected in Table 1. The AG group is colored in yellow, whereas the CTRL group is blue.

Our primary analysis was to investigate 12 brain regions of interest (ROIs) that have previously been shown to be susceptible to changes during vestibular stimulation from either HDT or spaceflight^3,25,27,35^. Those ROIs included the: left middle temporal gyrus, left insula, left post-central region, left superior frontal region, left middle frontal region, right angular gyrus, right rolandic operculum, right insula, right pre-central gyrus, right cerebellar lobule VI, and the left and right human vestibular cortex, identified to be the repective parietal operculum 2 region (OP2; Table 1). In our ROI analyses, we identified a significant group by time interaction in the right cerebellar lobule VI (Table 1; Fig. 2). That is, CTRL subjects on average showed a decrease in cerebellar activation during vestibular stimulation, whereas the AG group showed no change from pre- to late-HDT. Further, we identified group differences in the L superior frontal, R pre-central gyrus and L OP2 ROIs, where the AG group had less deactivation throughout all time points, including pre-HDT.

**Table 1:** ROI based analysis

	HDT + AG		Group		HDT		Age		Sex	
ROI	*β*	*p*	*β*	*p*	*β*	*p*	*β*	*p*	*β*	*p*
L Middle Temporal	−0.001	0.896	−0.053	0.680	0.003	0.143	−0.004	0.504	0.063	0.896
L Insula	0.001	0.532	−0.156	0.079	−0.001	0.632	−0.001	.930	0.046	0.524
L Post-central	−0.012	0.199	−0.232	0.543	0.001	0.935	−0.138	0.378	−0.412	0.200
L Superior Frontal	−0.001	0.842	−0.280	**0.042**	0.001	0.641	0.001	0.843	−0.052	0.630
L Middle Frontal	−0.005	0.143	−0.013	0.923	0.001	0.773	0.001	0.846	0.046	0.687
R Angular Gyrus	−0.001	0.670	−0.021	0.857	0.001	0.731	−0.002	0.701	−0.012	0.905
R Rolandic Operculum	0.001	0.593	−0.238	0.108	−0.003	0.427	0.005	0.504	−0.050	0.713
R Insula	0.002	0.670	−0.267	0.143	−0.002	0.300	−0.014	0.080	−0.009	0.952
R Pre-central Gyrus	0.002	0.468	−0.294	**0.044**	−0.001	0.691	0.005	0.468	−0.115	0.277
L OP2	0.005	0.191	−0.342	**0.046**	−0.002	0.408	−0.001	0.881	−0.076	0.571
R OP2	0.006	0.183	−0.109	0.650	−0.003	0.329	−0.003	0.818	−0.201	0.368
R Lobule VI	−0.005	**0.037**	0.067	0.518	0.001	0.647	−0.005	0.229	−0.099	0.247

Values that are significant are bolded under the effect. ROI is the region of interest that is being assessed in each respective column. HDT + AG refers to the group by time interaction of artificial gravity impacting the effects of HDT. HDT refers to the effects of time spent in the HDT environment. Beta values are listed under *β* to inform direction and strength of the relationship, while statistical values are listed under *p.* R and L are Right and Left, respectively.

### ROI brain-behavior correlations

**Fig. 3:**
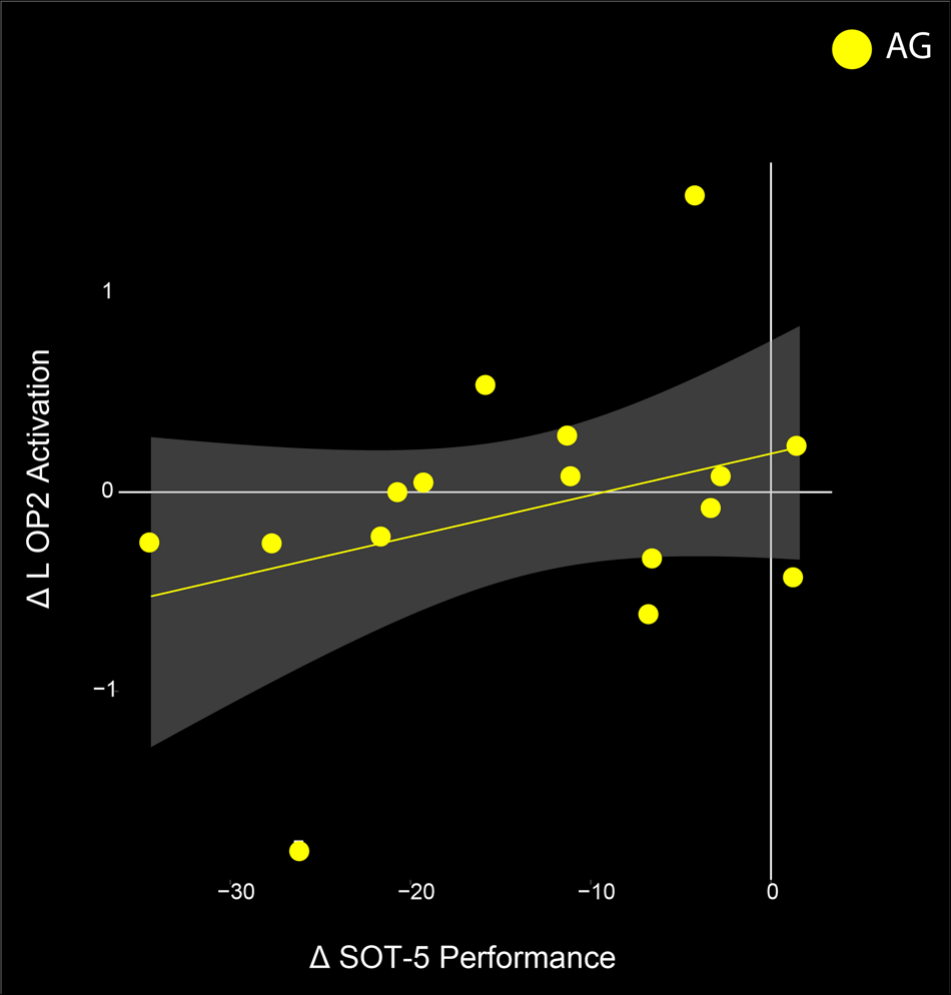
Brain and Behavior correlation. Change in the L OP2 ROI significantly correlates with change in the SOT-5 task performance from pre-to late-HDT in the AG group. The two most extreme data points do not qualify as statistical outliers. The gray indicates 95% confidence interval.

The functional mobility and balance data are more fully presented in a separate publication^17^; here we use the same behavioral data with the ROI results to investigate brain-behavior change-change correlations. We identified a significant change-change correlation in the AG group’s L OP2 activation and their SOT-5 performance (Fig. 3; *p* = 0.019, *t* = 2.6911). Those that showed the least decreases in activation in this region at HDT-58, compared to BDC-7, showed less pre to post HDT balance declines (SOT-5 measure).

### Exploratory whole brain HDT + AG

**Fig. 4:**
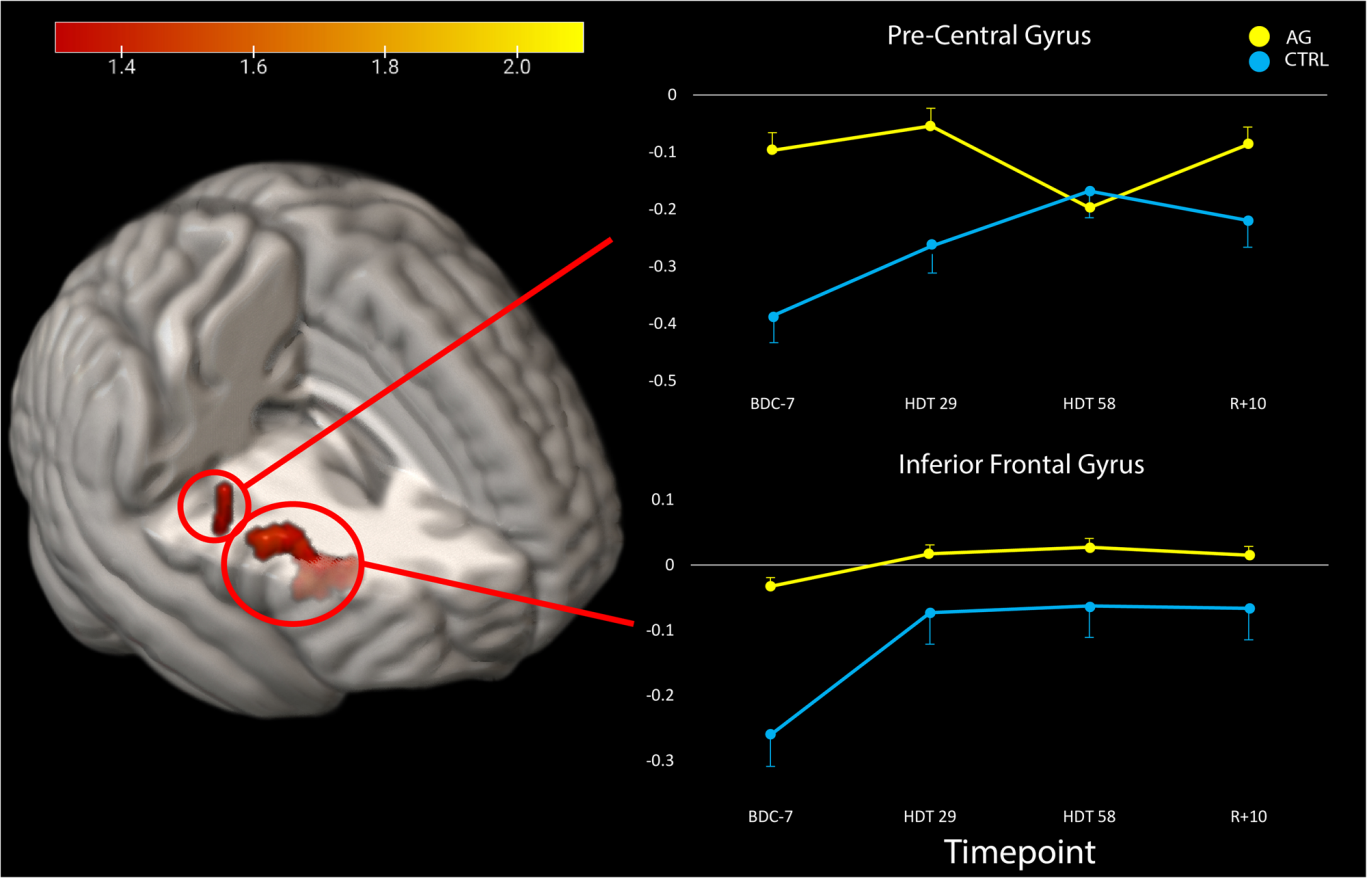
Group differences in activation after entering the HDT environment. The AG group showed an overall lack of change in HDT, whereas the CTRL group showed increased activation in both the pre-central gyrus and the inferior frontal gyrus.

We identified two clusters in the cerebral cortex that significantly differed in their activation profiles from pre- to post-HDT between groups (*p*_FWE-corr_ < 0.05, cluster size *k* > 5; Table 2). In these clusters, we identified that the AG group showed no statistical changes in activation after entering the HDT environment. However, the CTRL group decreased deactivation in both regions after entering HDT. In the precentral gyrus, the CTRL group showed initial increases in activation that continued to increase until HDT-58, where it then reached a plateau. In the inferior frontal gyrus, the CTRL participants had a large initial increase in activation that remained elevated throughout HDT and even through the recovery phase (Fig. 4). In both of these regions, the AG group showed relatively stable levels of activation.

**Table 2:** Regions that exhibited group by time effects

	TFCE Level		MNI Coordinates (mm)		
	*p*_FWE-corr_	Extent (k_E_)	*X*	*Y*	*Z*
Inferior Frontal Gyrus, R	0.032	235	48	26	10
Precentral Gyrus, R	0.041	50	54	2	30

Significance set at *p*_FWE-corr_ < 0.05, FWE corrected and *k* > 5. Clusters were labeled based on the AAL atlas.

*AG* artificial gravity, *HDT* head down tilt, *R* right.

## Discussion

This study investigated the use of AG to serve as an integrated countermeasure to mitigate HDT induced declines in vestibular processing and balance. We identified two clusters of brain activity exhibiting group (AG vs CTRL) by time interactions, in the precentral gyrus and the inferior frontal gyrus (Fig. 4, Table 2). We also investigated 12 ROIs that are active in response to vestibular stimulation and that exhibited changes in other HDT studies. Within these ROIs we identified three group main effects, and a group by time interaction in the right cerebellar lobule VI. In cerebellar lobule VI, activity remained stable throughout HDT for the AG group whereas the CTRL group decreased cerebellar activity during HDT and then recovered towards baseline levels post-HDT (Fig. 2, Table 1). Further, we found that those in the AG group that maintained their activation in the left OP2 region the most when entering HDT had the smallest decreases in balance from pre- to post-HDT (measured on the SOT-5 test, Fig. 3). Overall, these findings suggest that AG can mitigate some of the increased neural activation of vestibular processing typically associated with HDT, and indicate that it may be associated with balance performance. We suggest that AG should be further explored as an integrated countermeasure.

To assess our primary hypothesis we performed ROI analyses, extracting activation patterns from twelve ROIs that we have shown in prior studies to be activated in response to vestibular stimulation and to change with HDT. Of the twelve ROIs, one in the right cerebellar lobule VI showed a significant group by time interaction. In this ROI, the AG group’s activation remained stable after they entered the HDT environment, but the CTRL group displayed decreasing deactivation after entering HDT. This region is typically deactivated under this condition, meaning that it has less than zero “activity”. This deactivation is suppressed here, meaning that the signal is increased, but it is not reaching levels above zero. CTRL activation returned to baseline levels following the exit of the HDT period (Fig. 2). Lobule VI has previously been shown to be active during performance of complex sensorimotor tasks, and it shows topographical differences in hand versus foot movements^37^. Further, this region has also been shown to be engaged during n-back working memory tasks in conjunction with lobule VII^38^. Lobule VI has also been shown to atrophy in patients that have cerebellar ataxia, neuropathy and vestibular areflexia (CANVAS), along with lobules VIIa and VIIb^39,40^, supporting that it plays a role in vestibular processing and balance. Anatomical lesions in this lobule, as well as in lobules V and VIIa, have also been shown to hinder vestibular compensation in patients that have had a cerebellar stroke^41^. Thus we speculate that the use of AG in this HDT campaign affects this region as part of a complex network that receives vestibular and somatosensory inputs and integrates them to perform vestibular mediated sensorimotor tasks.

To examine the potential functional consequences of the observed brain changes, we conducted a brain-behavior change-change correlation analysis with the 12 ROIs and mobility and balance measures collected during this campaign. In the AG group, we identified a significant correlation between pre- to late-HDT activation change in the left OP2 region and the change from pre- to post-HDT SOT-5 balance condition. Participants that received AG and maintained their pre-HDT levels of activation in this region showed the least balance declines after exiting HDT (Fig. 3). The OP2 region has been suggested to be the human vestibular cortex^35,42^. Importantly, the SOT-5 balance condition specifically engages vestibular processing. The visual system is perturbed (eyes closed) and proprioception is unreliable (sway referenced platform), driving an increased reliance upon the vestibular system. The findings here indicate that those that received AG and subsequently were able to maintain their pre-HDT levels of activation were also able to preserve their performance in this balance condition the most. This suggests that AG may be a successful counter-measure for balance in this spaceflight analog, however there are individual differences in the extent of its effectiveness.

To identify if additional regions outside of our pre-selected ROIs showed changes due to HDT and AG, we conducted an exploratory whole brain analysis. We found that by HDT-29, the CTRL group increased activation in both the pre-central gyrus and the inferior frontal gyrus when receiving vestibular stimulation. In the pre-central gyrus activation continued to increase until HDT-58, whereas the AG group had no changes by HDT-29, and slightly decreased activation by HDT-58. By R + 10, the CTRL group decreased activation towards their pre-HDT levels and the AG returned to their pre-HDT activation levels. In the inferior frontal gyrus region, the AG group on average showed no change, while the CTRL group maintained their increased activation inside of HDT and during the recovery period. The precentral gyrus plays a key role in voluntary movement execution^43,44^. We have previously identified that this region is activated via vestibular stimulation, and shows a steeper slope of activation change during 70 days of bedrest compared to non-HDT controls^27^. Moreover, we recently reported increased activation in the precentral gyrus in response to vestibular stimulation following long duration spaceflight^3^. This is comparable to what we observed here in the CTRL group, whereas AG mitigated the effect. This supports that AG effectively mitigates changes in brain vestibular activity that occur with HDT; due to the similar changes that occur with spaceflight, this finding suggests that AG may be an effective countermeasure to spaceflight induced changes as well.

In addition to the precentral gyrus, we identified increased activation within the right inferior frontal gyrus (rIFG). CTRLs showed an increase in activation upon entering HDT that persisted throughout the 60 days, and the following 10 days of recovery (Fig. 4). The rIFG region has been repeatedly shown to be connected to a variety of functions, including sensorimotor, but has a considerable role in response inhibition and attentional control^45^. Interestingly, this region is not frequently associated with vestibular function, however in patients with acute vestibular neuritis there has been an identified association where an increased degree of nystagmus is associated with increased regional cerebral glucose metabolism in the rIFG^46^. Further, recall of vestibular sensation has been shown to activate both the rIFG and the left IFG^35^, however the left IFG is more typically associated with vestibular function. It is possible that activation of the rIFG in this instance is indicative of compensatory recruitment of the contralateral IFG. However, the rIFG is also reliably shown to be associated with motor inhibition through a variety of lesion and brain stimulation studies^47–50^. Motor inhibition is a vital aspect of conducting human movement as it allows a person to suppress inappropriate actions (preventing oneself from a harmful action), interrupting current actions (releasing the gas pedal to brake) and plays a key role in movement disorders^51,52^. Increased activation within this region in the control subjects and specifically associated with HDT could suggest a negative effect or increased cost of HDT on motor inhibition, which in this context, could lead to increased risk form these environments.

During the AGBRESA campaign, NASA, ESA and the DLR investigated whether AG could serve as an integrated countermeasure to target physiological and neurological deficits induced by HDT that models spaceflight. The overall findings of this campaign have varied widely, but within the sensorimotor domains there have been promising findings. Multiple investigations have identified AG to have some positive effect on balance and vestibular function^17,33^. We have also identified that it may increase neural efficiency in sensorimotor adaptation tasks^34^. We found that participants performed the paced serial audition test better during centrifugation^17^, but 30 min daily of AG in a short arm centrifuge seems insufficient to counteract HDT-induced cognitive declines^53^. It is possible that AG primarily works on sensorimotor and vestibular functions, as we see here that it has some effects on specific brain regions and function. However, further investigation is needed to understand its mechanistic effects and individual differences in responsivity.

The study here has several limitations that should be considered when interpreting the findings. First, participants were randomly split between the three groups, however, in three of our 12 ROIs main effects of group were identified, suggesting that even prior to HDT there may have been some differences between the groups. We interpret this as due to chance, however our statistical modeling takes this into account by analyzing predicted changes from participant’s individual baselines. Second, the sample size is limited, making it more difficult to identify subtle group and individual differences. We attempted to increase our power by combining the continuous and intermittent AG groups to mitigate this. Future investigations should increase the sample size to increase power and interpretability. Third, the current HDT campaign was only 60 days, whereas ISS missions typically take around 180 days. This may result in only partial dysfunction in HDT compared to astronauts. Lastly, AG may require individualized dosing. Here, the rotational speed was calculated to create a similar level of g in the z axis at the CoM, however the amount of time each participant received this was standardized to 30 min. It is important to note that while AG creates 1gz at the center of mass, the level of gz created at the vestibular organ is less than that. Improved methods of centrifugation that can created 1gz at the vestibular organ may have increased effects.

Here, we investigated the use of AG as an integrated countermeasure to target vestibular brain changes that occur with HDT, a spaceflight analog. The AG group received 30 min per day for 60 days, resulting in no activation changes in a cerebellar ROI, as well as the right precentral gyrus and right IFG in an exploratory whole brain analysis. In contrast, the CTRL group showed activation changes specific to HDT in these regions. We also identified a brain and behavior change-change correlation in the left OP2 ROI, where those that received AG and maintained their pre-HDT levels of activation in this region showed the least balance declines from pre- to post-HDT. We interpret these findings to suggest that AG may increase sensory stimulation and could result in preserving vestibular system function, even in environments where this system’s function typically declines. These findings support that further investigation into AG as a HDT and spaceflight countermeasure should be conducted.

## Methods

### Participants

Twenty-four individuals (8F; all individuals: 33.3 ± 9.17 yrs, 174.6 ± 8.6 cm, 74.2 ± 10.0 kgs) were recruited to participate in this study. All participants were screened for tolerability of AG, according to the AG2 protocol^31^, prior to enrollment. Participants were also selected to be of similar age (24–55 years), sex and education range to astronauts. Exclusion criteria included cardiovascular disease history, medications, and smoking for 6 months prior to entering the experiment. They provided written informed consent prior to participating; the protocol was approved by the University of Florida and NASA Institutional Review Boards as well as the regional medical association (Ärztekammer Nordrhein). This study was a piece of the larger joint investigation conducted by NASA, the European Space Association (ESA) and the German Aerospace Center (DLR) to identify if AG could serve as an integrated countermeasure to target spaceflight induced functional changes. The entirety of the study was conducted at the DLR’s: envihab facility in Cologne, Germany. As with larger studies, participants engaged in various other experiments during this time; here, we will only discuss those relevant to this investigation. Participants were also given free time throughout the day on a fixed schedule where they could read, operate a computer or do as they see fit while remaining in the HDT position. We have previously published cognitive and sensorimotor brain and behavior data that were collected as part of this larger campaign^17,34^.

All participants experienced 60 days of HDT with their head 6° below their feet where they spent 24 h per day in HDT. They slept in this position, while being allowed to shift their bodies as long as they stayed in this position. Prior to and following the 60 days of HDT they performed baseline and recovery testing, respectively. During the two weeks prior to entering HDT, participants experienced AG twice (11 days and 4 days prior) and then were randomized into three groups. Two of the groups received centrifugally applied AG for 30 min daily, either applied in one continuous bout, or intermittently for 6 bouts that consisted of 5 min each with 3 min between each bout. The third group was a control group that received no AG; all three groups spent the same time period in HDT. We initially examined for statistical differences in brain activation between the two AG groups and found none; thus we pooled them together into a joint AG group. AG was applied via a short arm centrifuge at the: envihab facility where rotational speed was customized with each participant based on their center of mass (CoM) to generate 1 g in the *z* axis at their CoM and around 2gz at their feet. Max speed was reached following a ramp up/down that did not exceed 5° s^−2^ to reduce negative tumbling sensations that can arise from vestibular stimulation in this manner. The participants were instructed to keep their body as still as they could, but they were not restrained to enforce this.

**Fig. 5:**
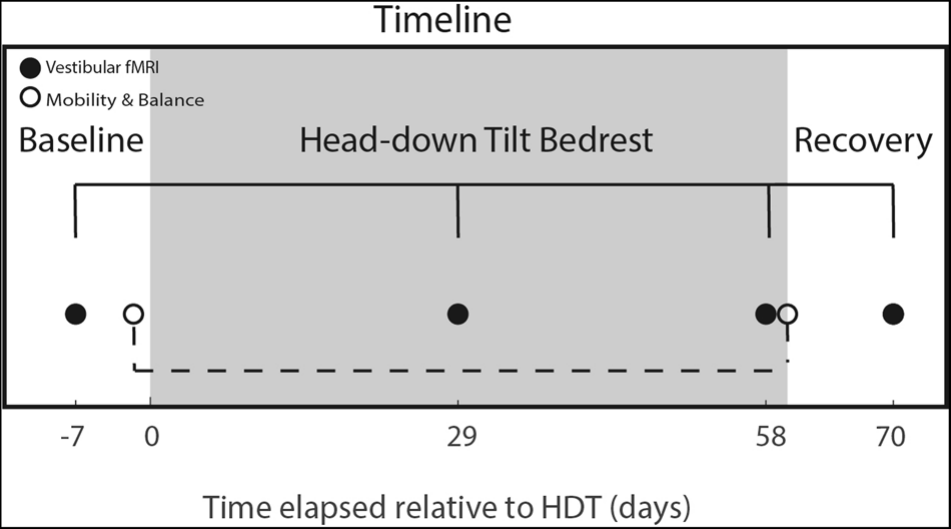
Task timeline. Functional MRI data were collected prior to HDT (7 days), twice during (29 and 58 days) and once following (10 days). Mobility and balance data for the brain-behavior correlation were collected one day prior to entering HDT and on the day participants exited HDT.

Here, we used a vestibular stimulation method, that was applied to the right side, and has been validated in healthy young adults, and used in both HDT^25,27^ and spaceflight studies^3^ while participants were in the MRI scanner. Vestibular stimulation was applied with a MRI compatible pneumatic tactile pulse system (Pn Tacticile Pulse System; PnTPS, Engineering Acoustics Inc.)^3,11,25,27,54^. This tapper works using compressed air to deliver low force taps (0.6 kg) to the lateral cheekbones. This has been repeatedly shown to elicit vestibular-evoked myogenic potentials (measured with electromyography, EMG), and to activate vestibular cortical regions and deactivate somatosensory and visual cortices (measured with fMRI), and to be less irritating to participants than other vestibular stimulation methods^54,55^. We measured brain activation during cheekbone taps compared with rest while participants were in the HDT position within an MRI scanner (described in further detail below). Participants underwent this stimulation four times (Fig. 5) for this experiment; 7 days pre-HDT (Baseline Data Collection (BDC) -7), 29 and 58 days in HDT (HDT29, HDT58) and 10 days post-HDT (recovery, R + 10).

### Mobility and balance measures

We included multiple measures of mobility and balance that have been shown to change with both spaceflight and HDT. This allowed us to test for brain-behavior correlations, to better understand the functional relevance of any observed brain changes.

The functional mobility test (FMT) is an obstacle course designed to require similar maneuvers as might be used to perform emergency egress from a space vehicle after landing^2^. It requires participants to move as quickly as possible, while walking, through a 6 m × 4 m obstacle course, making maneuvers around various foam pylons, under foam hurdles and between slalom bars. The first half is on a solid surface, whereas the participants walk on high density foam (Sunmate Foam, Dynamic Systems Inc. Leicester, NC, USA) for the second half to perturb proprioceptive inputs. The FMT has been shown to be sensitive for detecting mobility changes that occur with spaceflight^1,2,5,56^, and HDT^13,15,17,57^. The primary outcome measure is a participant’s completion time on the first trial of the obstacle course.

To assess balance, we utilized the Sensory Organization Test 5 and 5 M (SOT-5; SOT-5M) conducted with a computerized dynamic posturography system (Equitest, NeuroCom International, Clackamas, OR, USA). The SOT-5 required participants to remain in upright balance with their head upright, eyes closed and on a sway-referenced base. The SOT-5M consists of the same, however instead of maintaining their head in a rigid position they must perform dynamic head tilts of ±20°, at 0.33 Hz rate given via a metronome tone^58^ Participants performed 3 trials, each 20 s, and we used the median score. Equilibrium quotients (EQ) were calculated based on the peak-to-peak center of mass sway angle (*θ*), ${EQ}=100* [(1-(\theta /12.5^\circ )$]. The 12.5° represents the max theoretical limit of stability in the anterior-poster direction. Less sway during the task results in higher scores with a maximum of 100, interpreted as better performance. Note that both conditions are intended to assess one’s use of vestibular input for maintaining balance by depriving visual cues with eyes closed and disrupting proprioceptive feedback with the base of support that moves in proportion to body way.

### fMRI parameters

Vestibular stimulation was applied while fMRI data were acquired with a 3-Tesla Siemens Biograph MRI scanner located at the DLR’s: envihab facility. A gradient echo T2*-weighted echo-planar imaging sequence with the following parameters was used to acquire fMRI data: TR: 2500 ms, TE: 32 ms, flip angle: 90°, FOV: 192 × 192 mm, matrix: 64 × 64, slice thickness: 3.5 mm, voxel size: 3 × 3 × 3.5 mm, 37 slices. We also acquired a T1-weighted gradient-echo pulse sequence with the following parameters: TR: 1.9 s, TE: 2.4 ms, flip angle: 9°, FOV: 250 × 250 mm, matrix: 512 × 512, slice thickness: 1.0 mm, voxel size: 0.49 × 0.49 × 1.0 mm, 192 slices. The participants remained in the HDT position during the fMRI collection by lying on a foam wedge, however their head was flat within the head coil. Participants completed one fMRI run at each testing session. A run consisted of five, 24 s blocks of active tapping, with 20 s rest periods between each block. The taps were delivered at 1 Hz, with a total of 24 taps per block. Other fMRI tasks assessing visuomotor adaptation, dual-tasking and spatial work memory were conducted at the same time, but here we only focus on the vestibular fMRI.

### Whole brain fMRI Pre-processing

fMRI data were pre-processed using Statistical Parametric Mapping 12 (SPM12; version 7219)^59^, the Advanced Normalization Tools package (ANTs)^60^ and FSL command line tools^61^. This pre-processing pipeline is similar to what we have used in our past HDT work^25,34,62,63^. Field maps were created to identify and correct B0 inhomogeneities with the FSL topup tool^61^. Then, images were corrected for slice timing, then realigned and resliced to correct for volume-to-volume head motion in SPM12. We used the Artifact Detection Tool (ART; https://www.nitrc.org/projects/artifact_detect/) to identify volumes with framewise displacement motion ≥ 2.0 mm and global brain signal Z threshold ≥ 9; all outlier volumes were then statistically covaried during analysis. To move each run into a standard MNI space, we used multivariate templates created with ANTs^60^. First, for each participant we created a longitudinal T1 template across all time points with the AntsMultivariateTemplateConstruction.sh function. Next, using the same function we created participant specific fMRI multivariate templates with the fMRI data. These two templates were then co-registered using the AntsRegistration.sh function, and then the structural multivariate template was normalized to MNI space using the same function and an MNI152 template. The transformations created from these registrations were concatenated into a flow field and applied to the pre-processed fMRI images for each participant at each time point to bring their fMRI runs into standard space. Normalized images were then spatially smoothed with an 8 mm full width at half-maximum three dimensional Gaussian kernel.

### Cerebellar pre-processing

As in our previous analog and spaceflight fMRI work^3,25,34,62–64^, we used specialized pre-processing to remove and assess the cerebellum separately from the cerebral cortex. To do this, we used the CEREbellum Segmentation (CERES)^65^ pipeline and the Spatially Unbiased Infratentorial Template (SUIT)^66,67^. First, participant-specific T1 templates were uploaded to the CERES segmentation pipeline to isolate the cerebellum from the whole of the brain. The isolated cerebellum was then converted into a binary cerebellar mask from the CERES output using ImCalc in SPM12 and applied to each time point. Next, we used ANTs AntsRegistration.sh to transform the T1 cerebellar template into a standard space with the SUIT template. Then, each slice timed, realigned and resliced fMRI run was transformed to the participants T1 template space where it was masked with the individual’s cerebellar binary mask. Next, the masked fMRI data were transformed into SUIT template space using AntsApplyTransforms.sh. Finally, we applied a 2 mm full width at half-maximum three-dimensional smoothing Gaussian kernel to SUIT space cerebellar images in SPM12. We chose a 2 mm kernel here due to the small lobule size of the cerebellum, similar to other studies^66–68^.

### Mobility and balance behavioral statistical analyses

We used the nlme package^69^ in R 3.6.1^70^ to fit linear mixed effects models with restricted maximum likelihood (REML) estimation to examine mobility and balance performance changes over time. In each model we entered subject as a random intercept to allow for different starting points for each individual. We fit two models: (1) to evaluate the effects of the HDT and HDT + AG environment on performance and (2) to evaluate recovery after exiting the HDT and HDT + AG environment. These behavioral data were analyzed and presented in a previous published work^17^; the data are included here for brain-behavior correlation analyses.

### Subject-level fMRI statistics

For each individual subject, and at each time point, we calculated brain activation, and deactivation, on a voxel-by-voxel basis for vestibular stimulation versus rest. As in our previous longitudinal fMRI work, we set the first level masking threshold to –infinity^3,25,34,62–64^.

### Group level statistical analyses

Before testing for any group differences, we first verified that our pneumatic tapper was elicited the expected results. Next, to determine whether AG mitigated the effects of HDT we used multiple statistical models. Here, we assessed longitudinal changes comparing the groups in specific regions of interest (ROI). These ROIs were chosen because they showed activity changes in our prior HDT and spaceflight studies in response to vestibular stimulation^3,25,27^. In addition to this, we also selected the left and right parietal operculum 2 (OP2), as these regions have been identified to serve as a human vestibular cortex^35,36^. The parietal operculum is located between the inferior area of the postcentral gyrus and the posterior rami of the lateral fissure, within the posterior portion of this region. Then, we tested hypothesis-free longitudinal changes comparing subjects that received the centrifugal AG daily and the controls. Finally, we tested for brain-behavior correlations to evaluate whether participants’ activation changes across HDT were related to their balance and mobility performance changes.

In order to verify the pneumatic tapper method was working, we first tested the main effect of vestibular stimulation across all participants prior to entering the HDT environment at BDC-7. We set our statistical threshold at FWE < 0.05 while controlling for age and sex (Fig. 1).

We performed brain-behavior correlations to examine the association between any brain changes and changes to mobility and balance occurring with HDT and AG. Here, we assessed whether changes in brain activity from BDC-7 to HDT58 were related to changes in mobility and balance behavior from BDC-1 to R + 0 within our 12 pre-designated ROIs. We assessed this through Pearson correlation analysis conducted within R 3.6.1^70^.

### Whole brain time course of neural response to HDT + AG

To test the potentially mitigating effect of AG on HDT-induced changes, we implemented an a priori hypothesized weighted longitudinal model in a whole brain, exploratory manner. We created longitudinal contrasts that include pre-HDT time point BDC-7, HDT29 and HDT58 to investigate vestibular processing changes directly due to HDT. We also assessed recovery in these regions by assessing functional brain changes from HDT58 to R + 10, but only examining regions that changed due to HDT. The models were built using the Sandwich Estimator Toolbox for SPM12 (SwE)^71^, like what we have done in previous spaceflight analog investigations^3,25,27,34,62–64,72,73^. The SWE toolbox uses a noniterative marginal model to prevent within-subject convergence problems inherent to longitudinal designs, providing optimal analysis of longitudinal MRI data, especially with small data sets and missing data. The SwE default setup was used, modified only to use non-parametric wild bootstrapping with 999 permutations, which is recommended for small sample sizes^74^. Mean centered age and sex were included in the model as covariates. Significance was analyzed at a *p* < 0.05, family-wise error (FWE) corrected for multiple comparisons. For whole brain analysis, an explicit mask was used to investigate only gray matter effects in the cerebrum (and not the cerebellum, which was analyzed separately). This mask was created through binarizing the Computational Anatomy Toolbox 12 (CAT12)^75,76^ MNI-space gray matter template at a threshold of 0.1. Recovery was assessed only in regions that showed changes due to HDT by creating a results mask and implementing it with the recovery model. Cerebellar analyses were conducted only on the cerebellum as discussed above in “Cerebellar Pre-processing.”

## Data Availability

The datasets used and/or analyzed during the current study are available from the corresponding author on reasonable request. Moreover, the data can be accessed upon request to the NASA Life Sciences Data Archives.

## Code availability

The code used to analyze the current study is available from the corresponding author on reasonable request.

## References

[1] Tays GD (2021). The effects of long duration spaceflight on sensorimotor control and cognition. Front. Neural Circuits.

[2] Mulavara AP (2010). Locomotor function after long-duration space flight: effects and motor learning during recovery. Exp. Brain Res..

[3] Hupfeld KE (2022). Brain and behavioral evidence for reweighting of vestibular inputs with long-duration spaceflight. Cereb. Cortex.

[4] Boyle R (2021). Otolith adaptive responses to altered gravity. Neurosci. Biobehav. Rev..

[5] Mulavara AP (2018). Physiological and functional alterations after spaceflight and bed rest. Med. Sci. Sports Exerc..

[6] Cheron G (2006). Effect of gravity on human spontaneous 10-Hz electroencephalographic oscillations during the arrest reaction. Brain Res..

[7] Cheron G (2014). Gravity influences top-down signals in visual processing. PLoS ONE.

[8] Cebolla AM (2016). Cerebellar contribution to visuo-attentional alpha rhythm: insights from weightlessness. Sci. Rep..

[9] Demertzi A, Van Ombergen A, Tomilovskaya E (2016). Cortical reorganization in an astronaut’s brain after long-duration spaceflight. Brain Struct..

[10] Noohi F (2017). Functional brain activation in response to a clinical vestibular test correlates with balance. Front. Syst. Neurosci..

[11] Noohi F (2019). Deactivation of somatosensory and visual cortices during vestibular stimulation is associated with older age and poorer balance. PLoS ONE.

[12] Hargens AR, Vico L (2016). Long-duration bed rest as an analog to microgravity. J. Appl. Physiol..

[13] Reschke MF (2009). Postural reflexes, balance control, and functional mobility with long-duration head-down bed rest. Aviat. Space Environ. Med..

[14] Koppelmans V (2015). Exercise as potential countermeasure for the effects of 70 days of bed rest on cognitive and sensorimotor performance. Front. Syst. Neurosci..

[15] Koppelmans V (2017). Brain plasticity and sensorimotor deterioration as a function of 70 days head down tilt bed rest. PLoS ONE.

[16] Miller CA (2018). Functional task and balance performance in bed rest subjects and astronauts. Aerosp. Med. Hum. Perform..

[17] Tays GD (2022). The effects of 30 min of artificial gravity on cognitive and sensorimotor performance in a spaceflight analog environment. Front. Neural Circuits.

[18] Rubin AM, Liedgren SR, Odkvist LM, Larsby B, Aschan G (1979). Limb input to the cat vestibular nuclei. Acta Otolaryngol..

[19] Yates BJ, Jian BJ, Cotter LA, Cass SP (2000). Responses of vestibular nucleus neurons to tilt following chronic bilateral removal of vestibular inputs. Exp. Brain Res..

[20] Jian BJ, Shintani T, Emanuel BA, Yates BJ (2002). Convergence of limb, visceral, and vertical semicircular canal or otolith inputs onto vestibular nucleus neurons. Exp. Brain Res..

[21] MacDougall HG, Moore ST (2005). Marching to the beat of the same drummer: the spontaneous tempo of human locomotion. J. Appl. Physiol..

[22] Bles W, de Jong JM, de Wit G (1984). Somatosensory compensation for loss of labyrinthine function. Acta Otolaryngol..

[23] Horak FB, Hlavacka F (2001). Somatosensory loss increases vestibulospinal sensitivity. J. Neurophysiol..

[24] Carriot J, Jamali M, Cullen KE (2015). Rapid adaptation of multisensory integration in vestibular pathways. Front. Syst. Neurosci..

[25] Hupfeld KE (2020). Neural correlates of vestibular processing during a spaceflight analog with elevated carbon dioxide (CO_2_): a pilot study. Front. Syst. Neurosci..

[26] Mulder E (2014). Effects of five days of bed rest with and without exercise countermeasure on postural stability and gait. J. Musculoskelet. Neuronal Interact..

[27] Yuan P (2018). Vestibular brain changes within 70 days of head down bed rest. Hum. Brain Mapp..

[28] Clément GR (2020). Challenges to the central nervous system during human spaceflight missions to Mars. J. Neurophysiol..

[29] Hargens AR, Bhattacharya R, Schneider SM (2013). Space physiology VI: exercise artificial gravity and countermeasure development for prolonged space flight. Eur. J. Appl. Physiol.

[30] Clément GR, Bukley AP, Paloski WH (2015). Artificial gravity as a countermeasure for mitigating physiological deconditioning during long-duration space missions. Front. Syst. Neurosci..

[31] Linnarsson D (2015). Effects of an artificial gravity countermeasure on orthostatic tolerance, blood volumes and aerobic power after short-term bed rest (BR-AG1). J. Appl. Physiol..

[32] Clément GR (2022). International standard measures during the AGBRESA bed rest study. Acta Astronaut..

[33] De Martino E (2021). Lumbar muscle atrophy and increased relative intramuscular lipid concentration are not mitigated by daily artificial gravity after 60-day head-down tilt bed rest. J. Appl. Physiol..

[34] Tays GD (2023). Daily artificial gravity is associated with greater neural efficiency during sensorimotor adaptation. Cereb. Cortex.

[35] zu Eulenburg P, Caspers S, Roski C, Eickhoff SB (2012). Meta-analytical definition and functional connectivity of the human vestibular cortex. Neuroimage.

[36] Lopez C, Blanke O, Mast FW (2012). The human vestibular cortex revealed by coordinate-based activation likelihood estimation meta-analysis. Neuroscience.

[37] Schlerf JE, Verstynen TD, Ivry RB, Spencer RMC (2010). Evidence of a novel somatopic map in the human neocerebellum during complex actions. J. Neurophysiol..

[38] Grodd W, Hülsmann E, Ackermann H (2005). Functional MRI localizing in the cerebellum. Neurosurg. Clin. N. Am..

[39] Rust H (2017). VEMPs in a patient with cerebellar ataxia, neuropathy and vestibular areflexia (CANVAS). J. Neurol. Sci..

[40] Infante J (2018). Cerebellar ataxia, neuropathy, vestibular areflexia syndrome (CANVAS) with chronic cough and preserved muscle stretch reflexes: evidence for selective sparing of afferent Ia fibres. J. Neurol..

[41] Baier B, Müller N, Rhode F, Dieterich M (2015). Vestibular compensation in cerebellar stroke patients. Eur. J. Neurol..

[42] Ibitoye RT (2022). The human vestibular cortex: functional anatomy of OP2, its connectivity and the effect of vestibular disease. Cereb. Cortex.

[43] Banker, L. & Tadi, P. Neuroanatomy, precentral gyrus. https://europepmc.org/books/nbk544218 (2019). 31334938

[44] Chouinard PA, Paus T (2006). The primary motor and premotor areas of the human. Cereb. Cortex. Neuroscientist.

[45] Hampshire A, Chamberlain SR, Monti MM, Duncan J, Owen AM (2010). The role of the right inferior frontal gyrus: inhibition and attentional control. Neuroimage.

[46] Bense S (2004). Metabolic changes in vestibular and visual cortices in acute vestibular neuritis. Ann. Neurol..

[47] Aron AR, Behrens TE, Smith S, Frank MJ, Poldrack RA (2007). Triangulating a cognitive control network using diffusion-weighted magnetic resonance imaging (MRI) and functional MRI. J. Neurosci..

[48] Ditye T, Jacobson L, Walsh V, Lavidor M (2012). Modulating behavioral inhibition by tDCS combined with cognitive training. Exp. Brain Res..

[49] Chambers CD (2006). Executive ‘brake failure’ following deactivation of human frontal lobe. J. Cogn. Neurosci..

[50] Stramaccia DF (2015). Assessing the effects of tDCS over a delayed response inhibition task by targeting the right inferior frontal gyrus and right dorsolateral prefrontal cortex. Exp. Brain Res..

[51] Ganos C, Rothwell J, Haggard P (2018). Voluntary inhibitory motor control over involuntary tic movements. Mov. Disord..

[52] Duque J, Greenhouse I, Labruna L, Ivry RB (2017). Physiological markers of motor inhibition during human behavior. Trends Neurosci..

[53] Basner M (2021). Continuous and intermittent artificial gravity as a countermeasure to the cognitive effects of 60 days of head-down tilt bed rest. Front. Physiol..

[54] Wackym PA (2012). Rapid cVEMP and oVEMP responses elicited by a novel head striker and recording device. Otol. Neurotol..

[55] Brantberg K, Westin M, Löfqvist L, Verrecchia L, Tribukait A (2009). Vestibular evoked myogenic potentials in response to lateral skull taps are dependent on two different mechanisms. Clin. Neurophysiol..

[56] Cohen HS, Kimball KT, Mulavara AP, Bloomberg JJ, Paloski WH (2012). Posturography and locomotor tests of dynamic balance after long-duration spaceflight. J. Vestib. Res..

[57] Lee JK (2019). Head down tilt bed rest plus elevated CO_2_ as a spaceflight analog: effects on cognitive and sensorimotor performance. Front. Hum. Neurosci..

[58] Wood SJ, Paloski WH, Clark JB (2015). Assessing sensorimotor function following ISS with computerized dynamic posturography. Aerosp. Med. Hum. Perform..

[59] Penny, W. D., Friston, K. J., Ashburner, J. T., Kiebel, S. J. & Nichols, T. E. *Statistical Parametric Mapping: The Analysis of Functional Brain Images*. (Elsevier, 2011).

[60] Avants BB (2011). A reproducible evaluation of ANTs similarity metric performance in brain image registration. Neuroimage.

[61] Jenkinson M, Beckmann CF, Behrens TEJ, Woolrich MW, Smith SM (2012). FSL. Neuroimage.

[62] Salazar AP (2020). Neural working memory changes during a spaceflight analog with elevated carbon dioxide: a pilot study. Front. Syst. Neurosci..

[63] Salazar AP (2021). Visuomotor adaptation brain changes during a spaceflight analog with elevated carbon dioxide (CO_2_): a pilot study. Front. Neural Circuits.

[64] Salazar, A. P. et al. Changes in working memory brain activity and task-based connectivity after long-duration spaceflight. *Cereb. Cortex*10.1093/cercor/bhac232 (2022). 10.1093/cercor/bhac232 PMC10016051 35704860

[65] Romero JE (2017). CERES: A new cerebellum lobule segmentation method. Neuroimage.

[66] Diedrichsen J (2006). A spatially unbiased atlas template of the human cerebellum. Neuroimage.

[67] Diedrichsen J, Balsters JH, Flavell J, Cussans E, Ramnani N (2009). A probabilistic MR atlas of the human cerebellum. Neuroimage.

[68] Diedrichsen J (2011). Imaging the deep cerebellar nuclei: a probabilistic atlas and normalization procedure. Neuroimage.

[69] Pinheiro, J., Bates, D., DebRoy, S. & Sarkar, D. R Core Team. nlme: linear and nonlinear mixed effects models. R package version 3.1−148. R Foundation for statistical computing (2020).

[70] R Core Team. *R*: *A language and environment for statistical computing*. (R Foundation for Statistical Computing: Vienna, Austria, https://www.R-project.org/ 2019.

[71] Guillaume B (2014). Fast and accurate modelling of longitudinal and repeated measures neuroimaging data. Neuroimage.

[72] Yuan P (2016). Increased brain activation for dual tasking with 70-days head-down bed rest. Front. Syst. Neurosci..

[73] McGregor HR, Lee JK, Mulder ER, De Dios YE (2021). Brain connectivity and behavioral changes in a spaceflight analog environment with elevated CO_2_. Neuroimage.

[74] Guillaume, B. & Nichols, T. Non-parametric inference for longitudinal and repeated-measures neuroimaging data with the wild bootstrap. *Poster presented at the Organization for Human Brain* (2015).

[75] Dahnke R, Yotter RA, Gaser C (2013). Cortical thickness and central surface estimation. Neuroimage.

[76] Gaser & Kurth. Manual computational anatomy toolbox-CAT12. *Structural brain mapping Group at the Departments of* (2017).

